# Anger provocation increases limbic and decreases medial prefrontal cortex connectivity with the left amygdala in reactive aggressive violent offenders

**DOI:** 10.1007/s11682-018-9945-6

**Published:** 2018-08-25

**Authors:** Nicolette Siep, Franca Tonnaer, Vincent van de Ven, Arnoud Arntz, Adrian Raine, Maaike Cima

**Affiliations:** 1 Department of Clinical Psychological Science, Maastricht University, P.O. Box 616, 6200 MD Maastricht, The Netherlands; 2 Department of Research, Forensic Psychiatric Centre de Rooyse Wissel, Venray, The Netherlands; 3 Department of Cognitive Neuroscience, Maastricht University, Maastricht, The Netherlands; 4 Department of Clinical Psychology, University of Amsterdam, Amsterdam, The Netherlands; 5 Departments of Criminology, Psychiatry, and Psychology, University of Pennsylvania, Pennsylvania, PA USA; 6 Department of Developmental Psychopathology, Behavioural Science Institute, Radboud University Nijmegen, Nijmegen, The Netherlands; 7 Conrisq Group, Juvenile Youth Institutions (YouthCarePLUS), BJ Brabant, OGH Zetten & Pactum, Zetten, The Netherlands

**Keywords:** Impulsive aggression, Emotion regulation, Resting-state fMRI, Functional connectivity, Amygdala seed

## Abstract

Neurobiological models propose reactive aggression as a failure in emotion regulation, caused by an imbalance between prefrontal cortical control and excessive bottom-up signals of negative affect by limbic regions, including the amygdala. Therefore, we hypothesize a negative correlation between PFC and amygdala activity (pre/post resting-state scans) in violent offenders. In this study resting-state fMRI was administered before and after an emotion (anger and happiness) provocation or engagement task within 18 male violent offenders scoring high on reactive aggression, and 18 male non-offender controls. Research in emotional pre/post resting-state showed altered connectivity by task performance. Therefore, bilateral amygdala region of interest (ROI) whole brain functional connectivity analysis tested dynamic change differences between pre and post resting-state connectivity between groups. Self-reported anger showed a positive significant relationship with medial prefrontal cortex activity in the pre-task scan and significantly increased during the emotion task in both the violent and control group. Imaging results showed a significant decrease in amygdala – medial prefrontal functional connectivity in the violent offenders and an increase in the non-offender controls after the emotion task. The opposite pattern was found for amygdala connectivity with the (para) limbic regions: violent offenders showed increased connectivity and non-offender controls showed decreased connectivity. The present results indicate that reactive aggression might stem from a focus on emotion processing, as indicated by an increase in limbic functional connectivity. The combination of a focus on emotion, along with a lack of medial prefrontal cortex regulation, has the potential to grow out of control e.g. in reactive aggression.

Violence is a significant problem in our society. Reactive aggression, as opposed to proactive aggression, has been reported to be one of the major causes of violent behavior (World Health Organisation 2007).

Functional magnetic resonance imaging (fMRI) and especially resting state functional connectivity analysis might help in unraveling the ‘default mode network’ of reactive aggression (Shen 2015). Resting state functional connectivity focusses on the functional organization of both healthy and abnormal brain activity while ‘in rest’. Research conducting resting-state scans pre and post an emotional experimental task, testing for brain differences in functional connectivity has shown that pre and post resting state connectivity can be altered by fMRI task performance (Tung et al. 2013).

Emotion regulation research (Kohn et al. 2014) based on the theoretical framework of Gross and Ochsner (Gross 1998; Ochsner et al. 2012), suggests that the prefrontal cortex is the core brain structure in the neural network of cognitive emotion regulation (Kose et al. 2015; Marxen et al. 2016). Research in offender populations linking the origin of reactive aggression to specific brain regions, indicate that a combination of decreased prefrontal activity along with increased, hyperactive limbic activity (amygdala) is related to reactive aggression (e.g. Blair 2012; Coccaro et al. 2016; da Cunha-Bang et al. 2017, 2018; Diano et al. 2017; Heesink et al. 2018; Marxen et al. 2016; McCloskey et al. 2016; Skibsted et al. 2017). And research in youths with conduct problems link callous-unemotional traits to aberrant amygdala activity as a risk factors for aggression (Cardinale et al. 2017) and adolescent antisocial behavior (Dotterer et al. 2017).

Empirical neuroimaging findings indicate that aggressive people have functional (Anderson and Kiehl 2012; Bobes et al. 2013; Raine et al. 1998) abnormalities in the PFC and limbic structures, especially the amygdala (Buades-Rotger et al. 2017). Research on maladaptive emotion regulation shows a negative correlation between limbic regions, with a main focus on the amygdala, and the PFC (Coombs 3rd et al. 2014; McCloskey et al. 2016; Quaedflieg et al. 2015; Roy et al. 2009; Varkevisser et al. 2017). Maladaptive emotion regulation can take two forms: 1) under-regulation, which refers to the inability to contain emotional experiences sufficiently to engage in goal-directed behavior, and 2) over-regulation, which occurs when emotion regulation strategies are used to consistently stop emotion experience from unfolding (Greenberg and Bolger 2001; Roberton et al. 2014). At the present, much of the aggression research has focused on the hypothesized emotion under-regulation of anger, and seems to support this theory by findings of prefrontal hypo-metabolism and prefrontal serotonergic hypoactivity (New et al. 2002; Siever et al. 1999).

However, to our knowledge, no resting-state study has yet investigated the hypothesized abnormalities in functional connectivity between limbic structures and the PFC in reactive aggressive offenders, independent of any comorbid mental disorder. Moreover, the use of resting state scans before and after an emotion task gives the opportunity to investigate whether the negative correlation between limbic regions and the PFC (Coombs 3rd et al. 2014; McCloskey et al. 2016; Quaedflieg et al. 2015; Roy et al. 2009; Varkevisser et al. 2017) is related to maladaptive under-regulation or over-regulation in violent offenders.

Violent offenders are characterized by extreme aggressive behaviour (Grochowska and Kossowska 2012), with past aggression as a risk factor for violent recidivism (Mooney and Daffern 2011, 2015). Therefore, we investigate a paradigm that is able to provoke anger (Tonnaer et al. 2017), resulting in reactive aggression (Blair 2012). We focused on anger engagement, because anger is the emotional drive or motive behind reactive aggression (Averill 1983). As the reactive aggression diathesis is generally interpreted as a breakdown in amygdala - PFC connectivity causing under-regulation, it was predicted that emotion engagement (including anger) would decrease amygdala resting-state connectivity with the PFC in the violent offender group, compared to non-offender, age- and education matched controls.

## Methods

### Participants

The violent offenders (VOF, *N* = 19), hereafter referred to as the violent group, were recruited from an incarcerated male population at *Forensic Psychiatric Centre de Rooyse Wissel* (FPC dRW; Venray, the Netherlands), who were convicted for a violent crime (e.g. (attempted) manslaughter or murder, property crime with violence, bodily harm, domestic violence). The non-offender control group (NOC, *N* = 18), was recruited from a participant database and consisted of male participants with no history of violent behavior, matched on age, education level, and left/right handedness with the violent group. Participants were excluded if they had MRI contraindications, had an IQ below 80, reported psychotic symptoms, or were younger than 18 years. Exclusion criteria for the NOC included major neurological disorders, history of brain injury, current psychiatric disorders or substance abuse. Demographic and clinical characteristics of the sample are summarized in Table 1 with exception of one violent offender who was discarded because of poor fMRI data quality (see fMRI data preprocessing section).

**Table 1:** Demographic and clinical characteristics of the sample

	VOF *N* = 18	NOC *N* = 18	*t*-test *p* value	df	χ2 *p* value
Age, *M* (*SD*)	35.17 (7.12)	37.06 (15.24)	.64	34	–
Education, *N* (%)				3	
Primary	15 (83.33)	10 (55.56)	–		.07
Secondary	1 (5.56)	4 (22.22)	–		.15
College	2 (11.11)	3 (16.67)	–		.63
Axis-I disorder, *N* (%)				2	
Alcohol abuse	4 (22.22)	0	–		.03
Alcohol dependence	5 (27.780)	0	–		.02
Substance dependence	13 (72.22)	0	–		< .001
Depressive episode past	6 (33.33)	1 (5.56)	–		.04
PTSD	8 (44.44)	0	–		< .001
Axis-II disorder, *N* (%)				2	
Antisocial PD	11 (61.11)	0			< .001
Borderline PD	3 (16.67)	0			.07
Paranoid PD	3 (16.67)	0			.07

*PTSS* posttraumatic stress disorder, *PD* personality disorder

Psychopathy Checklist-Revised (PCL-R, Hare 2003) data was collected for a subsample of *N* = 12 VOF of which data was available. PCL-R total scores in general ranging from 0 to 40, ranged in the current sample from 12 to 28 (M = 21.2, SD = 6.2, showing that this is a sample displaying psychopathy characteristics). However, using the PCL-R as a diagnostic tool for the assessment of psychopathy (Acheson 2005; Lynam and Gudonis 2005, p 383), with scoring 30 or above indicating the diagnosis of psychopathy (Cooke et al. 2004) indicates no diagnostic psychopathic individuals within the tested sample of violent offenders.

### Procedure

**Fig. 1:**
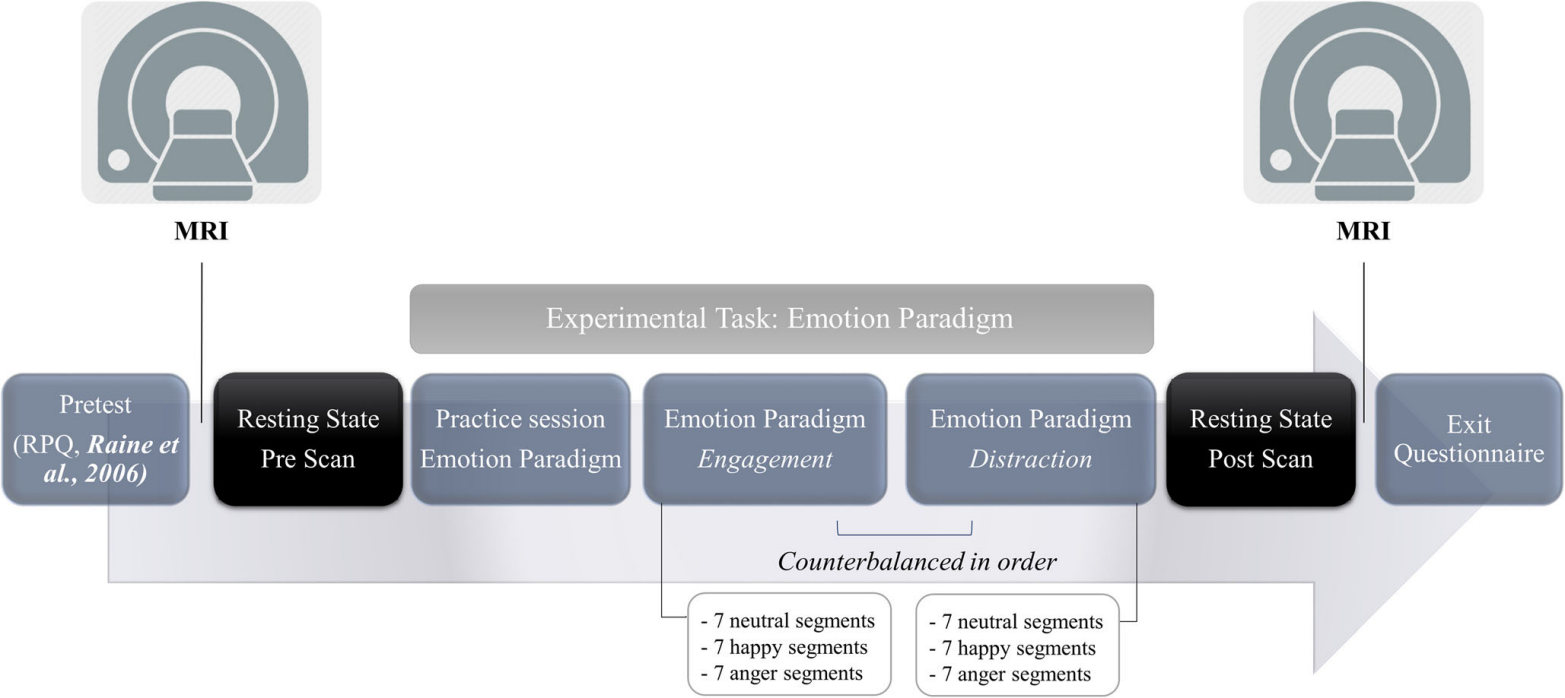
Procedure

Participants first completed the Reactive-Proactive Questionnaire (RPQ, Raine et al. 2006). Then, the fMRI scan started with a resting-state scan (see Fig. 1). After the first resting-state scan, participants completed an emotion engagement or distraction task (counterbalanced in order). After the first run of the emotion task an anatomical scan was started, during which the participant was instructed to relax. Then the second run of the emotion task was started. Immediately after the second run of the emotion task, the second resting-state fMRI scan was started. After the scanning session, the participant completed an exit-questionnaire.

### Measures

#### Experimental paradigm (Tonnaer et al. 2017)

Two 6 min resting-state scans were administered, one before and one after an emotional provocation or engagement (anger and happy) and distraction paradigm. Participants were instructed to lie still, relax and keep their eyes open. An adapted MRI version of the Anger Articulated Thoughts during Simulated Situations (ATSS) paradigm (Davison et al. 1995) was utilized in order to elicit anger and happy provocation and regulation. The current study focuses on data of the resting-state scans pre and post the experimental paradigm, testing for brain differences in functional connectivity as previous research has shown that pre and post resting state connectivity can be altered by fMRI task performance (Tung et al. 2013). FMRI data results on the experimental task, along with a more detailed description of the paradigm, its instructions and the immediate effects are reported elsewhere (Tonnaer et al. 2017). In this task participants were presented with audio audiotaped (anger, neutral and happy) stories, each within an *Engagement* condition instructing participants to focus on one’s emotional feeling, and a *Distraction* condition instructing participants to distract themselves from the presented stories during fMRI scanning. The order of these stories was randomized per participant. Each story was divided into seven segments of 15–20 s. At the end of each segment there was a tone, followed by a silence of 15 s. During this silence the participant either focused on one’s emotional (emotion engagement), or tried to distract himself from whatever he was feeling or thinking at that moment (emotion distraction). The order of the engagement and distraction task was randomized per participant and counterbalanced. The silence was followed by a visual analogue scale (VAS; 9 s), which participants used to rate the emotion intention at that moment (0 = *very happy* and, 100 = *very angry*).

The two anger stories were especially designed to elicit cognitions and feelings of anger. One anger story tells how the participant’s colleagues are spreading untrue rumors; the principal actor gets reprimanded by the boss because of these rumors; that one of these colleagues harasses the principal actor on the way home from work; the principal actor gets involved in a car crash because of this and is unrightfully blamed for the crash. The other anger story tells how the principal actor is waiting for the train, which is delayed, to go to the club. In the train the principal actors` partner is harassed by a drunken man and other people behave obnoxious. In the club it is impossible to order drinks and the man from the train punches the principal actors’ partner in the face. The happy stories include situations about the weather being sunny, being in love and winning the lottery. The neutral situations are about going grocery shopping or going through the routine of a normal workday.

#### Reactive-proactive aggression questionnaire (RPQ, Raine et al. 2006)

To validate the expected difference in reactive aggression between the VOF and the NOC, the Reactive-Proactive Aggression Questionnaire (Raine et al. 2006) was administered. The RPQ consists of 23 items: 11 items that measure reactive (i.e. impulsive) aggression and 12 items that measure proactive (i.e. instrumental) aggression, rated on a 3-point likert scale (0 = *never* and 2 = *always*). The internal reliability coefficients of the RPQ reactive and proactive-subscales are *α* = .81, and *α* = .84 respectively (Raine et al. 2006).

#### Exit-questionnaire

The exit-questionnaire consisted of 100 mm visual analogue scale, assessing representing variables that might influence resting-state functional connectivity other than the intended emotion engagement-distraction manipulation. These variables included physical discomfort inside the scanner (0 = *no physical complaints* and*,* 100 = *a lot of physical complaints*), nervousness (0 = *very nervous* and, 100 = *not nervous at all*), disturbance by the scanner noise (0 = *no annoyance at all* and, 100 = *very annoying*) or concentration problems (0 = *could not concentrate* and, 100 = *could concentrate very well*). In addition, participants were asked about difficulty (0 = *very difficult*, and 100 = *not difficult at all*) and success (0 = *not at all successful*, and 100 = *very successful*) in performing the emotion engagement and distraction tasks.

#### Image acquisition

Anatomical images were acquired using a Magnetom Allegra 3 T scanner (Siemens Healthcare, Netherlands) located at the Faculty of Psychology and Neuroscience, Maastricht University with a T1-weighted gradient echo (196 slices, TR = 2250 ms, TE = 26 ms, flip angle = 90°, field of view = 256 mm and voxel dimensions 1 × 1 × 1 mm). T2*-weighted functional measurements were acquired using a standard echo-planar imaging (EPI) sequence. Repetition time (TR) was set at 2000 ms, TE 30 ms, flip angle = 90°, 32 slices, 180 volumes, 3 × 3 × 3 mm. A slice tilt correction of −30 degrees was used to minimize inhomogeneity artefacts (Weiskopf et al. 2006).

### fMRI data preprocessing

Data preprocessing and analyses were conducted with Brainvoyager QX v2.4 (Brain Innovations, Maastricht, The Netherlands). The first two volumes of the functional images were omitted due to magnetization artifacts. Preprocessing of the remaining functional data included slice time correction using sinc interpolation, 3D motion correction using sinc interpolation, spatial smoothing (Gaussian kernel with full-width-at-half-maximum of 4 mm) and temporal filtering (linear trend removal). Individual functional datasets were then co-registered with structural images of the same participant and subsequently normalized using the Talairach and Tournoux transformation procedure (Talairach and Tournoux 1988). A quality check of functional brain coverage indicated that one of the violent offenders had insufficient amygdala coverage. This participant was removed from further analysis. Talairach coordinates were subsequently transformed to Montreal Neurological Institute (MNI) coordinates based on the ICBM−152 brain template (Lancaster et al. 2007) and all coordinates reported in this article are MNI coordinates. Individual volume time course (VTC) datasets were then averaged to create a group-based VTC functional brain mask to exclude voxels belonging to the outside of the functional brain.

### Resting-state functional connectivity analysis

**Fig. 2:**
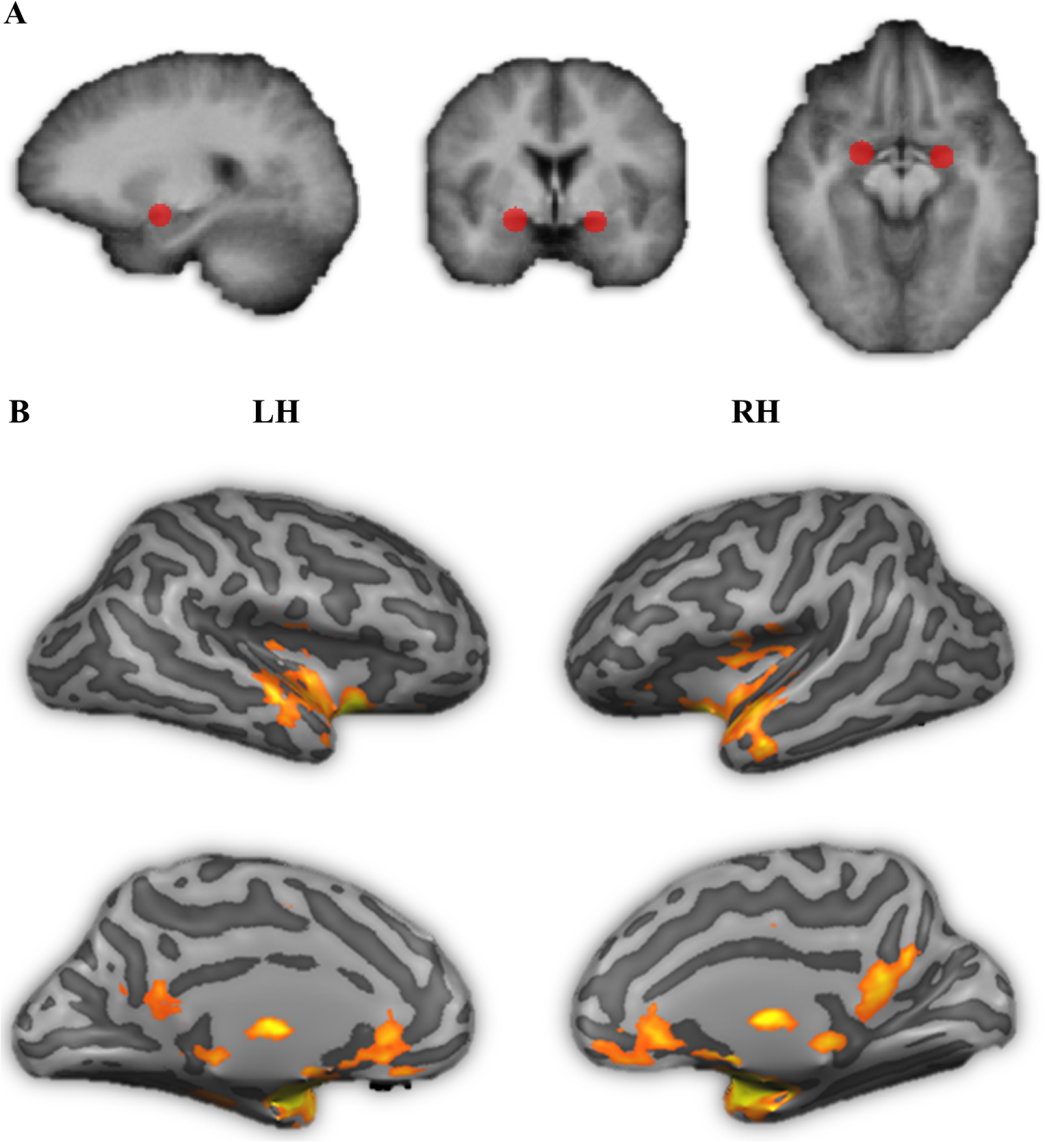
Panel A: A 6 mm sphere of the amygdala seeds used in the functional connectivity analysis were centered around the coordinates (MNI: × = −20, y = −4, z = −15) left, and (MNI: × = 22, y = −2, z = −15) right. Panel B: Bilateral amygdala seeds based functional connectivity mask, including posterior cingulate cortex, insula, medial prefrontal cortex, anterior temporal cortex with inclusion of the amygdala

This study used a mixed design with group (VOF vs. NOC) as between-subject factor and two fMRI runs (pre and post emotion task) as within subject factor (see the method sections ‘Image acquisition’ and ‘fMRI data preprocessing’ for more detailed information). In order to determine amygdala functional connectivity before and after the emotion task, an amygdala seed based connectivity analysis was performed, with amygdala seed MNI coordinates selected following the results of previous amygdala seed resting-state studies (Baeken et al. 2014; Cisler et al. 2013, Fig. 2). The seed regions were two 6-mm-diameter spheres designed to encompass the left and right amygdala (Fig. 2). In order to perform the analysis in Brainvoyager QX, first single study design matrix (SDM) files were created for each run of each participant using the NeuroElf matlab toolbox (www.neuroelf.net) and a custom-written resting state analysis toolbox developed at Maastricht University (Oertel-Knochel et al. 2013; van de Ven et al. 2013). These SDM files included the BOLD time series of the left and right amygdala seed regions and 122 *z*-normalized nuisance confound regressors: six 3D head motion parameters (x, y, z translations and x, y, z rotations) and their first derivatives, mean signal from the ventricles and white matter, global signal, and modelled oscillations at a frequency above.1 Hz (sine-cosine pairs). These SDM files were then used to run a random effects (RFX) GLM to find voxels that showed significant correlations with the left and right amygdala seeds separately, for each run of each participant.

The strategy of separate left versus right amygdala seed analysis was chosen due to the often reported strong correlational activity found between bilateral brain structures, resulting in multicollinearity and therefore reducing reliability when entering these two independent variables into the same model. To confirm this assumption we performed a post-hoc correlation analysis on the left and right amygdala seeds, which indeed showed significant correlations before (*r* = .61, *p* < .001) and after (*r* = .60, *p* < .001) the emotion task between the two seeds.

The Pearson correlation coefficients (*r*) maps resulting from the RFX GLM analysis were transformed using Fisher’s *R*-to-*Z* transformation (*Z* = .5 Ln [(1 + *R*)/(1-*R*)]), which yields variates that are approximately normally distributed. Then an *F*-group map was created, showing all voxels that had a significant correlation with the amygdala seeds at a conservative false discovery rate (FDR) of.001 for all participants over all runs. This map was transformed into a bilateral amygdala seed functional connectivity network mask (Fig. 2), which was used to *limit* the final main and group x time effects.

In the final analysis, a repeated measures ANOVA analysis with group (VOF vs. NOC) as a between subject factor and emotion task (pre/post) as a within-subject factor was conducted for the left and the right amygdala seed separately, to test for the hypothesized difference in amygdala connectivity. The resulting two *F*-maps were thresholded at a *p* value of.01 and a cluster size threshold of 108 mm^3^ (4 voxels) as determined by the calculation of a minimum cluster which protected against false positive clusters at 5% after 1000 Monte Carlo simulations (Forman et al. 1995). The Talairach coordinates of the peak voxels of the resulting functional regions of interest (ROIs) of these maps were converted into MNI space. Post-hoc analyses of simple effects within and between groups within these ROIs were corrected for multiple comparisons using Bonferroni.

#### Data availability

The datasets generated during and/or analyzed during the current study are available from the corresponding author on reasonable request.

## Results

### Self-report data

#### Reactive-proactive aggression questionnaire (RPQ, Raine et al. 2006)

Results indicated a significant difference (*p* < .001) between the reactive aggression score of the VOF (*M* = 14.67, *SD* = 5.17) and the NOC (*M* = 5.17, *SD* = 3.43), with only one control participant falling within the 1 SD range of the VOF. These results confirmed the expectation of higher reactive aggression in the VOF. The VOF also scored significantly higher on proactive aggression than the NOC (NOC, *M* = 1.00, *SD* = 1.61; VOF, *M* = 7.62, *SD* = 4.74; *p* < .001). However, analysis of group differences in proactive aggression while controlling for reactive aggression (i.e. ANCOVA) showed that group differences in proactive aggression were no longer significant (*p* = .07). On the contrary, group differences in reactive aggression were still significant when controlling for proactive aggression (*p* = .004), indicating that differences in reactive aggression in this study are primary.

#### Exit-questionnaire

On average there were no self-reported differences between the groups for physical discomfort (VOF, *M* = 22.11, *SD* = 27.60; NOC, *M* = 28.47, *SD* = 25.96; *p* = .49) and concentration (VOF, *M* = 72.24, *SD* = 24.05; NOC, *M* = 77.74, *SD* = 19.18; *p* = .47). There was a trend for a difference in nervousness, with the NOC scoring higher than the VOF (VOF, *M* = 65.24, *SD* = 34.63; NOC, *M* = 82.59, *SD* = 17.43; *p* = .08). The VOF reported being significantly more disturbed by the scanner noise (VOF, *M* = 55.00, *SD* = 33.37; NOC, *M* = 30.76, *SD* = 17.43; *p* = .02). Both groups indicated that the task was not too difficult, with no significant difference between the groups for the emotion engagement (VOF, *M* = 73.35, *SD* = 27.68; NOC, *M* = 69.35, *SD* = 29.94; *p* = .69) and the emotion distraction task (VOF, *M* = 89.18, *SD* = 10.02; NOC, *M* = 87.53, *SD* = 15.86; *p* = .72). However, the VOF did report that they expected to be significantly less successful in performing the emotion engagement (VOF, *M* = 64.88, *SD* = 33.27; NOC, *M* = 84.29, *SD* = 15.52; *p* = .04). Difference in expected success between groups was almost significant for the emotion distraction task, again with the VOF expected to be significantly less successful in performing the emotion distraction (VOF, M = 57.76, SD = 31.66; NOC, M = 77.41, SD = 22.36; *p* = .05). Further, no participants reported falling asleep. Pearson correlation coefficients analysis on the exit-questionnaire data and scan results indicate a significant negative correlation between disturbance by scanner noise and the superior temporal gyrus (*r* = −.37, *p* < .05), only for the pre resting state scan.

#### Anger

**Fig. 3:**
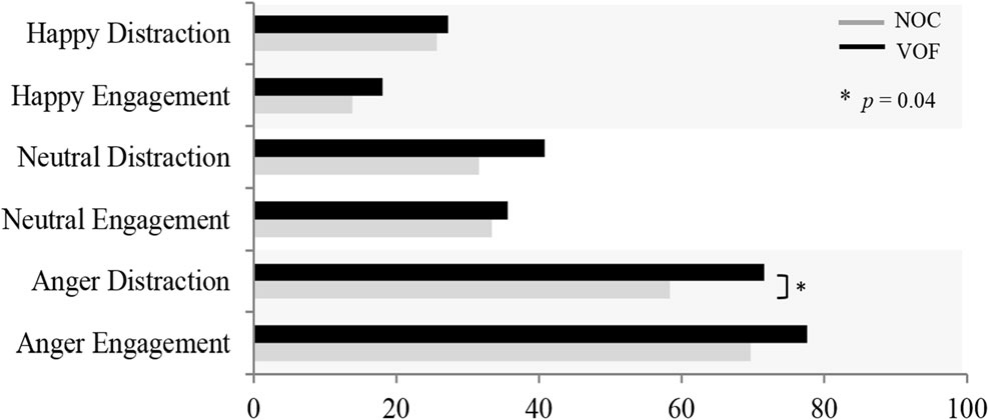
Mean VAS rating per group during ATSS. *P* value uncorrected for multiple comparisons

During the emotion task participants were asked to rate their experienced emotional state by means of a visual analogue scale (VAS; 0 = *very happy* and, 100 = *very angry*). Results for both groups (Fig. 3) indicated that anger was increased during the anger engagement segments compared to the neutral and happy story segments (both *ps* < .001). Results were similar for changes in self-reported anger during the anger distraction task (both *ps* < .001). An independent-samples *t*-test (uncorrected) indicated that there was a significant difference in self-reported anger between the two groups, with the NOC reporting less anger after the anger segments while distracting, compared to the VOF (VOF, *M* = 71.64, *SD* = 13.12*; p* = .04; NOC, *M* = 58.34, *SD* = 22.73).

### fMRI results

#### Right amygdala connectivity

The mixed effects ANOVA, with group as the between subject factor and emotion task performance as the within-subject factor, for the right amygdala seed did not reveal any significant results.

#### Left amygdala connectivity

**Fig. 4:**
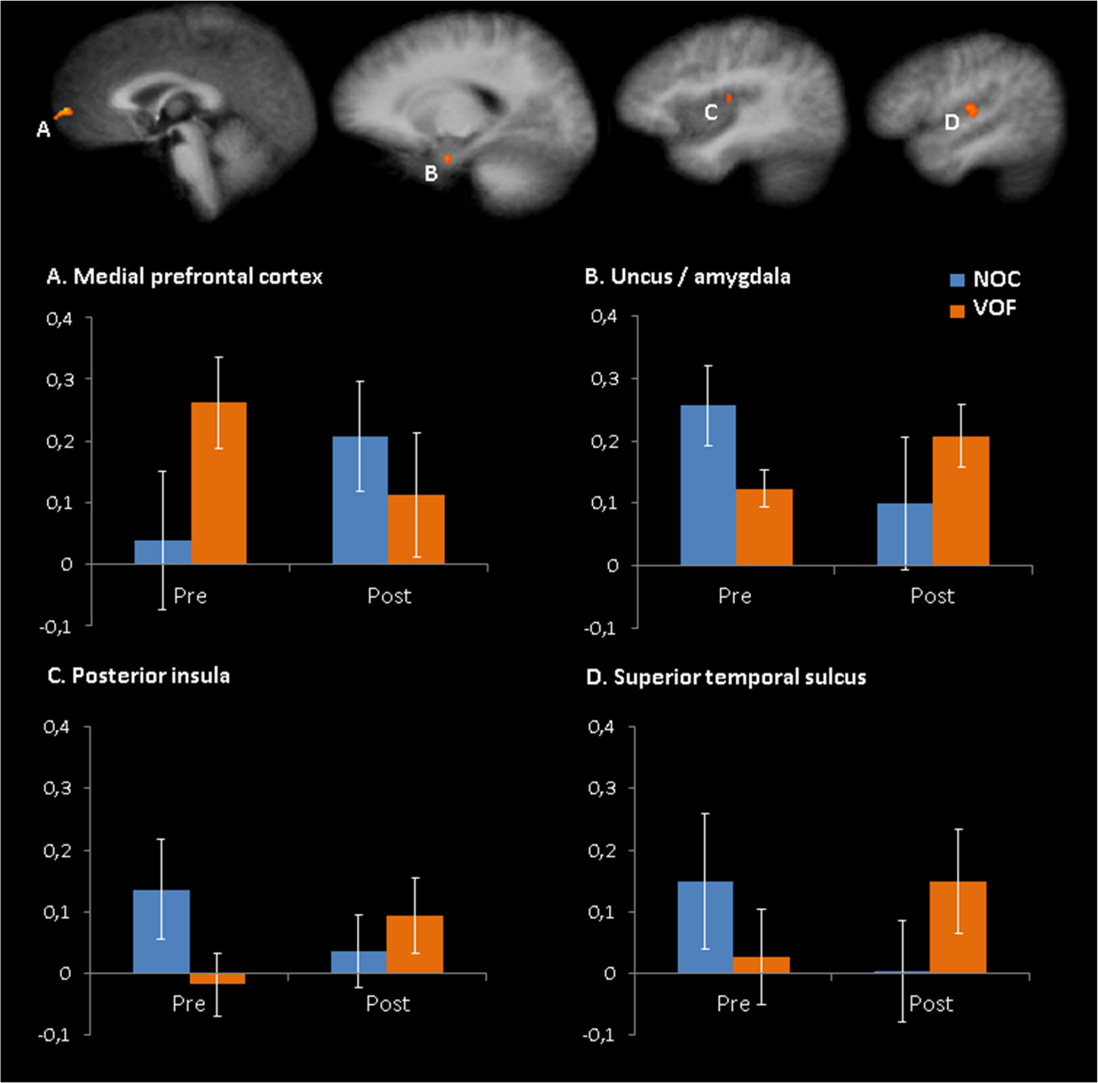
Significant group (VOF vs. NOC) × emotion provocation/distraction (pre vs. post) interactions. The y-axis of the graphs depicts the mean z values of functional connectivity of the left amygdala seed within each region. Error bars depict ±2 SE

Four ROIs with significant group × emotion engagement interactions were identified (Fig. 4, Table 2), including the left medial PFC, the left uncus/amygdala, the right posterior insula and the right superior temporal gyrus. In line with our hypothesis, there was a significant decrease in left medial PFC connectivity with the left amygdala seed from pre- to post-task in the VOF (*p* = .002), whereas the NOC showed a significant increase in left medial PFC connectivity with the left amygdala seed (*p* = .006).

**Table 2:** ROI details identified for interaction between group and within subject factor

					Average connectivity change Fisher’s z		Cohen’s d (change within groups)	
Brain region	L/R	MNI (x,y,z)	F	*p* value^*^	VOF	NOC	VOF	NOC
Medial prefrontal cortex	L	−1, 64, −4	27.32	< .001	−.15	.17	−.76	.74
Uncus/amygdala	L	−20, −6, −27	14.81	< .001	.08	−.16	.51	−.80
Posterior insula	R	46, −9, 9	16.05	< .001	.11	−.10	.84	−.64
Superior temporal gyrus	R	52, −12, 9	17.55	< .001	.12	−.15	.66	−.70

*L/R* Left/Right, *Cohen’s d* Mean difference/pooled SD, ^*^df = 1

For the (para) limbic regions (i.e., the left uncus/amygdala, the right posterior insula and the right superior temporal gyrus) the opposite pattern was found; the VOF showed a significant pre- to post-task increase in left amygdala functional connectivity with the right posterior insula (*p* = .018) and right superior temporal gyrus (*p* = .022), but not the left uncus/amygdala (*p* = .18). The NOC showed a significant pre- to post-task decrease in left amygdala functional connectivity with the right posterior insula (*p* = .028), the right superior temporal gyrus (*p* = .014) and the left uncus/amygdala (*p* = .004).

Post-hoc simple-effects analysis revealed that the largest difference between groups were pre-task, with a larger left medial prefrontal connectivity with the left amygdala seed in the VOF compared to the NOC (*p* = .004). Post-task the difference in left medial prefrontal connectivity with the left amygdala seed between groups was no longer significant (*p* = .34). There were also significant pre-task differences in left amygdala functional connectivity between groups in the left uncus/amygdala (*p* = .006) and the right posterior insula (*p* = .006), and during the post-emotion engagement condition in the right superior temporal gyrus (*p* = .034).

#### Correlation baseline left amygdala and subjective measures

To investigate whether the significant difference between groups in self-reported noise hindrance and also nervousness (almost significant) related to group differences in left amygdala functional connectivity in the resulting ROIs, a correlation analysis was performed on self-reported scanner noise disturbance and nervousness with the change z-scores in amygdala functional connectivity (post-task minus pre-task) in the resulting ROIs. This analysis also did not reveal any significant correlation (all *r*s < .21; all *p*s > .24), indicating that changes in connectivity from pre- to post task cannot be explained by influences of noise disturbance or nervousness.

#### Correlation aggression and baseline resting scan results

Since post-hoc simple-effects analysis revealed that the largest difference between groups were pre-task, we performed correlation analyses between aggression questionnaires and the baseline resting scan results to explore whether differences in aggression might relate to general differences already reflected in the pre-task scan results. Results showed a significant relation between activity in the medial PFC connectivity during the pre-task scan and (reactive as well as proactive) aggression (*r* = .36, *p* < .05 for reactive aggression and *r* = .45, *p* < .01 for proactive aggression). Moreover, results showed a significant negative relation between reactive aggression and activity in the posterior insula and the amygdala during the pre-task scan (*r* = −.45, *p* < .01 for the posterior insula and *r* = −. 35, *p* < .05 for the amygdala) and a significant negative relationship between proactive aggression and activity in the superior temporal sulcus (*r* = −.42, *p* < .05).

## Discussion

The aim of the present resting-state fMRI study was to investigate brain connectivity using the amygdala as a region of interest (ROI) before and after an emotion task in reactive aggressive violent offenders (VOF, *N* = 18) versus non-offender controls (NOC, *N* = 18, age and education matched). We used resting-state scans pre and post an emotional task, testing for brain differences in functional connectivity as earlier research has shown that pre and post resting state connectivity can be altered by fMRI task performance (Tung et al. 2013). This emotion task required participants to listen to anger, happy, and neutral stories while paying attention to whatever they were feeling or thinking at that moment (i.e. anger engagement) or to distract themselves from whatever they were feeling or thinking at that moment (i.e. anger distraction). By measuring functional connectivity using the amygdala as a ROI, we investigate the inter-relationship of other brain regions simultaneously engaged with the amygdala in this emotion task (Rogers et al. 2007). Previous research on maladaptive emotion regulation has indicated that a combination of decreased prefrontal activity along with increased, hyperactive limbic activity (amygdala) is related to reactive aggression (e.g. Blair 2012; Coccaro et al. 2016; da Cunha-Bang et al. 2017, 2018; Diano et al. 2017; Heesink et al. 2018; Marxen et al. 2016; McCloskey et al. 2016; Skibsted et al. 2017).

In line with the study’s primary hypothesis results showed that left amygdala – medial PFC connectivity was decreased from pre- to post-emotion task in the VOF and increased in the control group. No changes in right amygdala functional connectivity were found. In addition, significant interactions were also found in (para) limbic regions, including the uncus/amygdala, posterior insula and superior temporal gyrus. These effects were opposite from that found in the medial PFC; the emotion task significantly increased amygdala connectivity with (para) limbic regions in the VOF and decreased connectivity in the NOC.

Interestingly, additional analysis indicated that group differences in amygdala – medial PFC were present only prior to the emotion task, with stronger connectivity in the VOF. It could be speculated that the pre-task scan differences reflect general differences in aggression as a correlational analysis between aggression questionnaires and pre-task scan activity showed a relationship between medial PFC functioning during the pre-task scan and (reactive as well as proactive) aggression. This is in line with earlier research showing a link between mPFC functioning or prefrontal cortical control and anger or aggression (Lotze et al. 2007; Potegal et al. 2010; Takahashi et al. 2014). Further, as it proposed that amygdala – PFC connectivity is the neurobiological correlate of emotion regulation (Ochsner and Gross 2005). This suggests that the VOF were regulating their emotions. However, the NOC did not show this connectivity pattern. In other words, VOF were regulating emotions in a situation in which the NOC did not find it necessary to do so. Therefore, these results can be interpreted as emotional over-regulation in the VOF. Emotional over-regulation occurs when an individual uses emotion regulation strategies in an effort to consistently stop the emotion experience from unfolding (Greenberg and Bolger 2001). Although speculative, current self-report measures suggest that the VOF might have attempted to hide, avoid or suppress signs of nervousness. In addition, previously it has been suggested that aggressive acts of the over-regulated people are typically of greater intensity, because a greater level of anger arousal is necessary to overcome the intense over-regulation strategies (Willner and Blackburn 1988). This proposition fits with the general observation of increased emotional intensity of reactive aggressive acts compared to that of proactive aggressive acts.

At first instance, the present additional indications of over-regulation might seem contradictory to previous findings of emotional under-regulation in reactive aggressive people. However, in our opinion, these findings are not necessarily incongruous and can be explained twofold. First, while using emotional avoidance or expressive suppression can be beneficial in certain situations (Butler et al. 2003), for example in macho-environment where emotions like sadness and anxiety are interpreted as signs of weakness, the constant use of these strategies require considerable effort and energy (Kashdan et al. 2006). If much of the available processing resources are used for continuous default over-regulation, this also means that less resources are available when more active executive control is required, e.g. when the person is provoked, resulting in emotion under-regulation. This hypothesis nicely fits with the passive paradigm used in this resting-state study which indicates overregulation already visible in the pre-test scans, compared to a more active neuroimaging paradigm (i.e. instructing participants to perform an executive functioning task), indicating under-regulation in aggressive people (Raine et al. 1998). A second explanation is that trait aggressiveness is higher in the VOF, requiring constant regulation. This argument is in line with the model by Megargee (later augmented by Blackburn (1993), which proposes that VOF can be classified into either the under-controlled type or the chronically over-controlled type. In this model the over-controlled type is inhibiting angry feelings until stress may exceed his ability to resist. As a result of provocation their defense breaks down and consequently, violent behavior occurs. Future studies should address these different hypotheses.

The mechanism of over-control of anger is also worked out in more recent cognitive theories (Denson et al. 2011; Rusting and Nolen-Hoeksema 1998), emphasizing the role of rumination regarding provocation. Indeed, the present results reported additionally that left amygdala functional connectivity with the superior temporal gyrus was increased after emotion engagement, in the VOF only. Increased activity in the superior temporal sulcus has been previously linked to rumination (Cooney et al. 2010), which may be broadly defined as a pattern of recursive thinking focused on one’s negative mood. Rumination is associated with the worsening of negative mood states, greater affective responding to negative material, and increased access to negative memories (for a review see Cooney et al. 2010). For example, self-reported rumination has been found to be associated with amygdala activation during the up-regulation of negative affect in healthy people, suggesting that an increased tendency to ruminate exacerbates the neural processing of negative information (Ray et al. 2005). Therefore, it is possible that reactive aggressive people are more likely to ruminate after being exposed to an emotionally challenging situation. In turn, this may lead to increased negativity bias (Cima et al. 2014; Lobbestael et al. 2013), making them more sensitive to negative stimuli and consequently and increasing the change of reactive aggressive behavior. This proposition is in line with neurobiological models of reactive aggression (Blair 2004; Davidson et al. 2000; Phelps et al. 2004; Raine and Yang 2006) stating that individuals with faulty regulation of negative emotions propose a serious risk for aggressive behaviour.

As this is, to our knowledge, the first study to examine the dynamics in amygdala functional connectivity in people with reactive aggressive behavior before and after an emotion engagement and distraction task. Present results should be interpreted with caution and replication studies are encouraged. In addition, the following limitation should be considered. Although both groups followed the exact same experimental procedure, the exit questionnaire revealed that the VOF were significantly more disturbed by the scanner noise. Interactions in amygdala resting-state functional could therefore also be attributed to other factors than the emotion engagement (e.g. time), which remains a general limitation of resting-state studies. However, the increased disturbance by the scanner noise might also be explained by a decrease in frustration tolerance, which seems characteristic for those with reactive aggressive behavior and therefore highly dependent. In addition, self-report of scanner disturbance and nervousness did not correlate with pre-task connectivity or pre- to post-task change scores in any of the implicated ROIs, and could therefore not explain present results. In addition, as the whole brain analysis revealed a network known to be involved in emotion regulation it is assumed that the present results can indeed be attributed to the emotion engagement. Moreover, the use of one VAS with anchors of very happy to very angry within the experimental task is unfortunate, given that these do not represent opposite poles of a single construct. In future research two VAS scales are needed indicating not at all happy to very happy, and not at all angry to very angry. Another limitation is the fact that although substance use is prohibited for all incarcerated offenders and regularly drug tests are executed randomly during their stay by means of breathalyzers and urine tests, no arrangements have been made in advance for standard substance use testing within the day of the fMRI testing session.

In conclusion, the current study investigated dynamic changes in amygdala connectivity resting-state functional connectivity in violent offenders showing reactive aggression, before and after an emotion (engagement and distraction) task. In line with neurobiological models of reactive aggression, results indicate that there is a decrease in amygdala – medial PFC functional connectivity in the VOF and an increase in the NOC. An opposite pattern was found in (para) limbic regions. Present results indicate that reactive aggression can be seen as resulting from a dominance of emotion processes, as indicated by an increase in limbic functional connectivity. This is especially problematic given the combination of emotion processing dominance along with a lack of medial prefrontal cortex regulation, leading to a loss of behavioral control when aroused with reactive aggression as a result. In addition, an increase in amygdala – superior temporal gyrus connectivity was found after the emotion task, which has been previously linked to rumination. Most treatment protocols regarding reactive aggression have focused on improving anger control with the underlying impression of decreasing violence. In line with the current finding it is suggested that treatment of reactive aggression should focus on rumination, acceptance, skills to handle anger expression and adaptive regulation of emotions.
